# Tris(ethyl­enediamine)zinc(II) dichloride monohydrate

**DOI:** 10.1107/S1600536808027979

**Published:** 2008-09-06

**Authors:** Lin Cheng, Yan-Yan Sun, Ya-Wen Zhang, Gang Xu

**Affiliations:** a Department of Chemistry and Chemical Engineering, Southeast University, Nanjing, People’s Republic of China; b Department of Chemistry and Chemical Engineering, State Key Laboratory of Coordination Chemistry, Nanjing University, Nanjing, People’s Republic of China

## Abstract

The asymmetric unit of the title compound, [Zn(C_2_H_8_N_2_)_3_]Cl_2_·H_2_O, contains a discrete [Zn(C_2_H_8_N_2_)_3_]^2+^ cation with a distorted octa­hedral geometry around Zn, two uncoordinated chloride ions and one water mol­ecule. The crystal structure exhibits N—H⋯O, N—H⋯Cl and O—H⋯O hydrogen bonds.

## Related literature

For related structures, see: Bernhardt & Riley (2003 ▶); Cernak *et al.* (1984 ▶); Emsley *et al.* (1989 ▶); Muralikrishna *et al.* (1983 ▶); Nesterova *et al.* (2006 ▶); Wu *et al.* (2001 ▶).
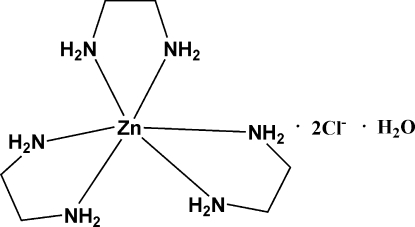


## Experimental

### 

#### Crystal data

[Zn(C_2_H_8_N_2_)_3_]Cl_2_·H_2_O
*M* *_r_* = 334.60
Monoclinic, 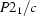

*a* = 8.8165 (10) Å
*b* = 11.9379 (14) Å
*c* = 14.4043 (17) Å
β = 92.804 (2)°
*V* = 1514.2 (3) Å^3^
*Z* = 4
Mo *K*α radiation
μ = 1.97 mm^−1^
*T* = 293 (2) K
0.25 × 0.22 × 0.16 mm

#### Data collection

Bruker APEX CCD diffractometer
Absorption correction: multi-scan (*SADABS*; Sheldrick, 2000 ▶) *T*_min_ = 0.639, *T*_max_ = 0.744
11550 measured reflections
2975 independent reflections
2511 reflections with *I* > 2σ(*I*)
*R*_int_ = 0.030

#### Refinement

*R*[*F* ^2^ > 2σ(*F* ^2^)] = 0.035
*wR*(*F* ^2^) = 0.085
*S* = 1.10
2975 reflections
145 parameters
H-atom parameters constrained
Δρ_max_ = 0.39 e Å^−3^
Δρ_min_ = −0.27 e Å^−3^

### 

Data collection: *SMART* (Bruker, 2000 ▶); cell refinement: *SAINT* (Bruker, 2000 ▶); data reduction: *SAINT*; program(s) used to solve structure: *SHELXS97* (Sheldrick, 2008 ▶); program(s) used to refine structure: *SHELXL97* (Sheldrick, 2008 ▶); molecular graphics: *SHELXTL* (Sheldrick, 2008 ▶); software used to prepare material for publication: *SHELXTL*.

## Supplementary Material

Crystal structure: contains datablocks I, global. DOI: 10.1107/S1600536808027979/bt2779sup1.cif

Structure factors: contains datablocks I. DOI: 10.1107/S1600536808027979/bt2779Isup2.hkl

Additional supplementary materials:  crystallographic information; 3D view; checkCIF report

## Figures and Tables

**Table 1:** Hydrogen-bond geometry (Å, °)

*D*—H⋯*A*	*D*—H	H⋯*A*	*D*⋯*A*	*D*—H⋯*A*
N1—H1*D*⋯Cl2^i^	0.90	2.86	3.739 (3)	165
N2—H2*C*⋯Cl1	0.90	2.50	3.363 (3)	162
N2—H2*D*⋯Cl2^ii^	0.90	2.48	3.332 (2)	158
N3—H3*C*⋯O1*W*^iii^	0.90	2.27	3.159 (3)	169
N3—H3*D*⋯Cl2	0.90	2.73	3.605 (3)	165
N4—H4*C*⋯O1*W*^iv^	0.90	2.39	3.260 (3)	164
N4—H4*D*⋯Cl1	0.90	2.52	3.375 (3)	159
N5—H5*D*⋯Cl2^i^	0.90	2.57	3.420 (3)	158
N6—H6*C*⋯Cl1^iv^	0.90	2.44	3.309 (3)	161
N6—H6*D*⋯Cl2	0.90	2.58	3.471 (3)	172
O1*W*—H1*WA*⋯Cl1	0.85	2.25	3.097 (3)	171
O1*W*—H1*WB*⋯Cl2^v^	0.85	2.34	3.187 (3)	180

Symmetry codes: (i) 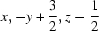; (ii) 
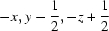
; (iii) 
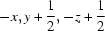
; (iv) 
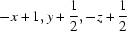
; (v) 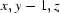.

**Fig. 1.**
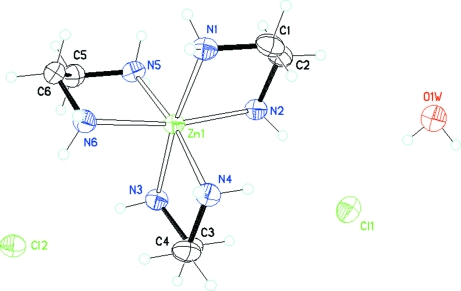
The asymmetric unit of the title compound with 30% displacement ellipsoids.

**Fig. 2.**
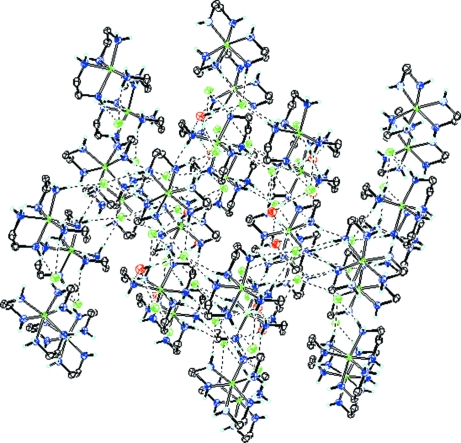
Partial packing diagram. The H atoms bonded to C atoms are omitted for clarity.

## supplementary crystallographic information

### Comment

The preparation of complexes including different stereoisomers is a fascinating and promising means. There are many complexes including [Zn(en)_3_]^2+^ cation (en = ethylenediamine), which have been reported, due that [Zn(en)_3_]^2+^ cation has two simple and intuitive stereoisomers (Bernhardt *et al.*, 2003; Cernak *et al.*, 1984; Emsley *et al.*, 1989; Muralikrishna *et al.*, 1983; Nesterova *et al.*, 2006; Wu *et al.*, 2001). Different from the similar compound [Zn(en)_3_]Cl_2_.2H_2_O (Muralikrishna *et al.*, 1983; Wu *et al.*, 2001), here, we report a salt [Zn(en)_3_]Cl_2_.H_2_O. In the asymmetric unit of the salt, there are only one crystal water molecule.

The asymmetric unit of the title salt, [Zn(en)_3_]Cl_2_.H_2_O, contains a discrete [Zn(en)_3_]^2+^ cation, two uncoordinated chloride ions and one water molecule. The Zn(II) ion displays a distorted octahedral geometry, being surrounded by three en ligands. The Zn···N distances are between 2.159 (2) and 2.220 (2) Å. Each en acts as a chelating bidentate ligand. In crystal, the [Zn(en)_3_]^2+^ cations, chloride ions and the crystal water are linked together by N—H···O,  N—H···Cl and O—H···Cl hydrogen bonds (Table 2).

### Experimental

To a solution of ZnCl_2_.2H_2_O (0.172 g, 1 mmol) in CH_3_OH (5 ml), an aqueous solution (5 ml) of bib (bib = 1,3-bis(4,5-Dihydro-1*H*-imidazol-2-yl)benzene) (0.214 g, 1 mmol) was added. After the mixture was stirred for half an hour, a white precipitate formed. 3 ml en was added to the mixture and the precipitate disappeared. Then the mixture was stirred for an hour and filtered. The filtrate was allowed to evaporate slowly at room temperature. After 3 weeks, colorless block shaped crystals were obtained in 40% yield (0.034 g) based on Zn(II).

### Refinement

H atoms were located in a difference map, but refined using a riding model with N—H = 0.90, O—H = 0.85 Å and C—H = 0.97 Å and with *U*_iso_(H) = 1.2 *U*_iso_(C,N,O).

### Crystal data

**Table:**

[Zn(C_2_H_8_N_2_)_3_]Cl_2_·H_2_O	*F*(000) = 704
*M**_r_* = 334.60	*D*_x_ = 1.468 Mg m^−^^3^
Monoclinic, *P*2_1_/*c*	Mo *K*α radiation, λ = 0.71073 Å
Hall symbol: -P 2ybc	Cell parameters from 783 reflections
*a* = 8.8165 (10) Å	θ = 2.5–28.0°
*b* = 11.9379 (14) Å	µ = 1.97 mm^−^^1^
*c* = 14.4043 (17) Å	*T* = 293 K
β = 92.804 (2)°	Block, colorless
*V* = 1514.2 (3) Å^3^	0.25 × 0.22 × 0.16 mm
*Z* = 4	

### Data collection

**Table:**

Bruker APEX CCD diffractometer	2975 independent reflections
Radiation source: fine-focus sealed tube	2511 reflections with *I* > 2σ(*I*)
graphite	*R*_int_ = 0.030
φ and ω scan	θ_max_ = 26.0°, θ_min_ = 2.2°
Absorption correction: multi-scan (SADABS; Sheldrick, 2000)	*h* = −10→10
*T*_min_ = 0.639, *T*_max_ = 0.744	*k* = −14→14
11550 measured reflections	*l* = −17→17

### Refinement

**Table:**

Refinement on *F*^2^	Primary atom site location: structure-invariant direct methods
Least-squares matrix: full	Secondary atom site location: difference Fourier map
*R*[*F*^2^ > 2σ(*F*^2^)] = 0.035	Hydrogen site location: inferred from neighbouring sites
*wR*(*F*^2^) = 0.085	H-atom parameters constrained
*S* = 1.10	*w* = 1/[σ^2^(*F*_o_^2^) + (0.0384*P*)^2^ + 0.5868*P*] where *P* = (*F*_o_^2^ + 2*F*_c_^2^)/3
2975 reflections	(Δ/σ)_max_ < 0.001
145 parameters	Δρ_max_ = 0.39 e Å^−^^3^
0 restraints	Δρ_min_ = −0.27 e Å^−^^3^

### Special details

**Table:**

Geometry. All e.s.d.'s (except the e.s.d. in the dihedral angle between two l.s. planes) are estimated using the full covariance matrix. The cell e.s.d.'s are taken into account individually in the estimation of e.s.d.'s in distances, angles and torsion angles; correlations between e.s.d.'s in cell parameters are only used when they are defined by crystal symmetry. An approximate (isotropic) treatment of cell e.s.d.'s is used for estimating e.s.d.'s involving l.s. planes.
Refinement. Refinement of *F*^2^ against ALL reflections. The weighted *R*-factor *wR* and goodness of fit *S* are based on *F*^2^, conventional *R*-factors *R* are based on *F*, with *F* set to zero for negative *F*^2^. The threshold expression of *F*^2^ > σ(*F*^2^) is used only for calculating *R*-factors(gt) *etc*. and is not relevant to the choice of reflections for refinement. *R*-factors based on *F*^2^ are statistically about twice as large as those based on *F*, and *R*- factors based on ALL data will be even larger.

### Fractional atomic coordinates and isotropic or equivalent isotropic displacement parameters (Å^2^)

**Table:**

	*x*	*y*	*z*	*U*_iso_*/*U*_eq_	
Zn1	0.24154 (3)	0.55616 (3)	0.20878 (2)	0.03553 (12)	
Cl1	0.31636 (9)	0.23340 (7)	0.36125 (6)	0.0567 (2)	
Cl2	0.20561 (8)	0.87644 (7)	0.38972 (5)	0.0505 (2)	
C1	0.3870 (3)	0.3760 (3)	0.1009 (2)	0.0555 (8)	
H1A	0.4452	0.3450	0.1538	0.067*	
H1B	0.4297	0.3480	0.0446	0.067*	
C2	0.2244 (4)	0.3402 (3)	0.1041 (2)	0.0540 (8)	
H2A	0.1681	0.3659	0.0486	0.065*	
H2B	0.2186	0.2591	0.1058	0.065*	
C3	0.1673 (4)	0.5327 (3)	0.4086 (2)	0.0507 (8)	
H3A	0.1431	0.4536	0.4034	0.061*	
H3B	0.1264	0.5610	0.4653	0.061*	
C4	0.3359 (4)	0.5484 (3)	0.4120 (2)	0.0557 (9)	
H4A	0.3601	0.6273	0.4192	0.067*	
H4B	0.3816	0.5085	0.4649	0.067*	
C5	0.0887 (4)	0.7490 (3)	0.1124 (2)	0.0565 (9)	
H5A	0.0391	0.7783	0.1659	0.068*	
H5B	0.0411	0.7824	0.0569	0.068*	
C6	0.2557 (4)	0.7787 (3)	0.1193 (2)	0.0508 (8)	
H6A	0.3040	0.7535	0.0640	0.061*	
H6B	0.2676	0.8594	0.1238	0.061*	
N1	0.3960 (3)	0.4984 (2)	0.10299 (17)	0.0465 (6)	
H1C	0.4885	0.5300	0.1105	0.070*	
H1D	0.3684	0.5262	0.0466	0.070*	
N2	0.1568 (3)	0.3873 (2)	0.18697 (16)	0.0419 (6)	
H2C	0.1778	0.3383	0.2333	0.063*	
H2D	0.0546	0.3854	0.1839	0.063*	
N3	0.0993 (3)	0.5935 (2)	0.32796 (16)	0.0443 (6)	
H3C	−0.0010	0.5790	0.3232	0.067*	
H3D	0.1079	0.6675	0.3389	0.067*	
N4	0.3974 (3)	0.5059 (2)	0.32603 (16)	0.0445 (6)	
H4C	0.4947	0.5276	0.3223	0.067*	
H4D	0.3872	0.4312	0.3204	0.067*	
N5	0.0720 (3)	0.6272 (2)	0.10862 (16)	0.0456 (6)	
H5C	−0.0218	0.6039	0.1214	0.068*	
H5D	0.0933	0.6075	0.0504	0.068*	
N6	0.3279 (3)	0.7250 (2)	0.20173 (16)	0.0444 (6)	
H6C	0.4288	0.7342	0.1982	0.067*	
H6D	0.3054	0.7678	0.2506	0.067*	
O1W	0.2475 (3)	0.0426 (2)	0.21973 (17)	0.0692 (7)	
H1WA	0.2630	0.1002	0.2536	0.104*	
H1WB	0.2361	−0.0020	0.2649	0.104*	

### Atomic displacement parameters (Å^2^)

**Table:**

	*U*^11^	*U*^22^	*U*^33^	*U*^12^	*U*^13^	*U*^23^
Zn1	0.03116 (18)	0.0416 (2)	0.03389 (18)	−0.00316 (13)	0.00251 (12)	0.00012 (13)
Cl1	0.0520 (5)	0.0563 (5)	0.0622 (5)	0.0042 (4)	0.0079 (4)	0.0108 (4)
Cl2	0.0449 (4)	0.0597 (5)	0.0467 (4)	0.0018 (3)	0.0009 (3)	−0.0014 (4)
C1	0.0461 (18)	0.073 (2)	0.0480 (18)	0.0078 (16)	0.0089 (14)	−0.0111 (17)
C2	0.0550 (19)	0.056 (2)	0.0503 (19)	−0.0051 (16)	0.0010 (15)	−0.0111 (16)
C3	0.0565 (19)	0.061 (2)	0.0348 (15)	0.0002 (16)	0.0076 (14)	−0.0003 (14)
C4	0.0529 (19)	0.073 (2)	0.0403 (17)	0.0025 (16)	−0.0075 (14)	−0.0069 (16)
C5	0.056 (2)	0.060 (2)	0.0534 (19)	0.0148 (16)	0.0015 (15)	0.0061 (16)
C6	0.067 (2)	0.0420 (18)	0.0440 (17)	−0.0034 (15)	0.0052 (15)	0.0025 (14)
N1	0.0359 (13)	0.0597 (17)	0.0444 (14)	−0.0059 (12)	0.0072 (10)	−0.0020 (12)
N2	0.0349 (12)	0.0484 (15)	0.0425 (13)	−0.0093 (11)	0.0032 (10)	−0.0006 (11)
N3	0.0368 (13)	0.0514 (15)	0.0453 (14)	−0.0006 (11)	0.0072 (10)	−0.0015 (12)
N4	0.0354 (13)	0.0527 (16)	0.0451 (14)	−0.0013 (11)	−0.0023 (10)	−0.0031 (12)
N5	0.0357 (13)	0.0582 (17)	0.0426 (13)	−0.0029 (11)	−0.0004 (10)	0.0052 (12)
N6	0.0407 (13)	0.0467 (15)	0.0461 (14)	−0.0083 (11)	0.0042 (11)	−0.0033 (12)
O1W	0.0747 (18)	0.0651 (17)	0.0678 (16)	0.0051 (12)	0.0032 (14)	0.0022 (12)

### Geometric parameters (Å, °)

**Table:**

Zn1—N6	2.158 (2)	C5—C6	1.513 (5)
Zn1—N2	2.167 (2)	C5—H5A	0.9700
Zn1—N5	2.196 (2)	C5—H5B	0.9700
Zn1—N1	2.203 (2)	C6—N6	1.467 (4)
Zn1—N4	2.208 (2)	C6—H6A	0.9700
Zn1—N3	2.220 (2)	C6—H6B	0.9700
C1—N1	1.464 (4)	N1—H1C	0.9000
C1—C2	1.498 (4)	N1—H1D	0.9000
C1—H1A	0.9700	N2—H2C	0.9000
C1—H1B	0.9700	N2—H2D	0.9000
C2—N2	1.472 (4)	N3—H3C	0.9000
C2—H2A	0.9700	N3—H3D	0.9000
C2—H2B	0.9700	N4—H4C	0.9000
C3—N3	1.472 (4)	N4—H4D	0.9000
C3—C4	1.496 (4)	N5—H5C	0.9000
C3—H3A	0.9700	N5—H5D	0.9000
C3—H3B	0.9700	N6—H6C	0.9000
C4—N4	1.466 (4)	N6—H6D	0.9000
C4—H4A	0.9700	O1W—H1WA	0.8500
C4—H4B	0.9700	O1W—H1WB	0.8503
C5—N5	1.463 (4)		
			
N6—Zn1—N2	168.95 (9)	H5A—C5—H5B	108.3
N6—Zn1—N5	80.73 (9)	N6—C6—C5	109.4 (2)
N2—Zn1—N5	92.59 (9)	N6—C6—H6A	109.8
N6—Zn1—N1	91.62 (9)	C5—C6—H6A	109.8
N2—Zn1—N1	80.16 (9)	N6—C6—H6B	109.8
N5—Zn1—N1	95.18 (9)	C5—C6—H6B	109.8
N6—Zn1—N4	94.68 (9)	H6A—C6—H6B	108.2
N2—Zn1—N4	93.19 (9)	C1—N1—Zn1	106.97 (18)
N5—Zn1—N4	170.26 (9)	C1—N1—H1C	117.9
N1—Zn1—N4	93.52 (9)	Zn1—N1—H1C	111.9
N6—Zn1—N3	93.60 (9)	C1—N1—H1D	109.7
N2—Zn1—N3	95.45 (9)	Zn1—N1—H1D	111.1
N5—Zn1—N3	92.20 (9)	H1C—N1—H1D	99.1
N1—Zn1—N3	171.56 (9)	C2—N2—Zn1	108.81 (18)
N4—Zn1—N3	79.46 (9)	C2—N2—H2C	106.0
N1—C1—C2	109.6 (3)	Zn1—N2—H2C	116.1
N1—C1—H1A	109.8	C2—N2—H2D	113.2
C2—C1—H1A	109.8	Zn1—N2—H2D	111.6
N1—C1—H1B	109.8	H2C—N2—H2D	100.9
C2—C1—H1B	109.8	C3—N3—Zn1	106.68 (18)
H1A—C1—H1B	108.2	C3—N3—H3C	109.1
N2—C2—C1	110.0 (2)	Zn1—N3—H3C	119.5
N2—C2—H2A	109.7	C3—N3—H3D	108.6
C1—C2—H2A	109.7	Zn1—N3—H3D	106.6
N2—C2—H2B	109.7	H3C—N3—H3D	106.0
C1—C2—H2B	109.7	C4—N4—Zn1	108.07 (17)
H2A—C2—H2B	108.2	C4—N4—H4C	110.2
N3—C3—C4	109.3 (3)	Zn1—N4—H4C	115.8
N3—C3—H3A	109.8	C4—N4—H4D	112.3
C4—C3—H3A	109.8	Zn1—N4—H4D	98.3
N3—C3—H3B	109.8	H4C—N4—H4D	111.7
C4—C3—H3B	109.8	C5—N5—Zn1	107.18 (17)
H3A—C3—H3B	108.3	C5—N5—H5C	113.0
N4—C4—C3	109.7 (2)	Zn1—N5—H5C	110.4
N4—C4—H4A	109.7	C5—N5—H5D	105.6
C3—C4—H4A	109.7	Zn1—N5—H5D	110.2
N4—C4—H4B	109.7	H5C—N5—H5D	110.2
C3—C4—H4B	109.7	C6—N6—Zn1	107.84 (18)
H4A—C4—H4B	108.2	C6—N6—H6C	107.0
N5—C5—C6	109.4 (2)	Zn1—N6—H6C	118.0
N5—C5—H5A	109.8	C6—N6—H6D	106.3
C6—C5—H5A	109.8	Zn1—N6—H6D	113.7
N5—C5—H5B	109.8	H6C—N6—H6D	103.4
C6—C5—H5B	109.8	H1WA—O1W—H1WB	95.1

### Hydrogen-bond geometry (Å, °)

**Table:**

*D*—H···*A*	*D*—H	H···*A*	*D*···*A*	*D*—H···*A*
N1—H1D···Cl2^i^	0.90	2.86	3.739 (3)	165.
N2—H2C···Cl1	0.90	2.50	3.363 (3)	162.
N2—H2D···Cl2^ii^	0.90	2.48	3.332 (2)	158.
N3—H3C···O1W^iii^	0.90	2.27	3.159 (3)	169.
N3—H3D···Cl2	0.90	2.73	3.605 (3)	165.
N4—H4C···O1W^iv^	0.90	2.39	3.260 (3)	164.
N4—H4D···Cl1	0.90	2.52	3.375 (3)	159.
N5—H5D···Cl2^i^	0.90	2.57	3.420 (3)	158.
N6—H6C···Cl1^iv^	0.90	2.44	3.309 (3)	161.
N6—H6D···Cl2	0.90	2.58	3.471 (3)	172.
O1W—H1WA···Cl1	0.85	2.25	3.097 (3)	171.
O1W—H1WB···Cl2^v^	0.85	2.34	3.187 (3)	180.

Symmetry codes: (i) *x*, −*y*+3/2, *z*−1/2; (ii) −*x*, *y*−1/2, −*z*+1/2; (iii) −*x*, *y*+1/2, −*z*+1/2; (iv) −*x*+1, *y*+1/2, −*z*+1/2; (v) *x*, *y*−1, *z*.
